# Implementation of Kidney Biopsy in One of the Poorest Countries in the World: Experience from Zinder Hospital (Niger)

**DOI:** 10.3390/jcm13030664

**Published:** 2024-01-24

**Authors:** Hassane Moussa Diongolé, Zeinabou Maiga Moussa Tondi, Abdoulazize Garba, Kabirou Ganiou, Laouali Chaibou, Djibrilla Bonkano, Illiassou Aboubacar, Abdoul Aziz Seribah, Abdoul Madjid Abdoulaye Idrissa, Akinfenwa Atanda, Lionel Rostaing

**Affiliations:** 1 Department of Nephrology and Dialysis, Université André Salifou de Zinder, Zinder B.P.656, Niger; diongolen@yahoo.fr (H.M.D.); garbaabdoulazize@gmail.com (A.G.); adeniyin@gmail.com (K.G.); 2 Hôpital National de Zinder, Zinder B.P.656, Niger; laoualichaibou@yahoo.fr (L.C.); az_seribah@yahoo.fr (A.A.S.);; 3 Department of Nephrology and Dialysis, Université Abdou Moumouni, Niamey 10662, Niger; zeinab_maiga@yahoo.fr; 4 Hospital National Amirou Boubacar Diallo Lamordé Niamey, Niamey 10146, Niger; bbdjibrilla@gmail.com; 5 Hôpital de Référence de Maradi, Maradi 12481, Niger; illiassouaboubacar@gmail.com; 6 Aminu Kano Teaching Hospital, Kano 700101, Nigeria; akinzo123@gmail.com; 7 CHU Grenoble, Néphrologie-Hémodialyse, Aphérèses-Transplantation Rénale, 38043 Grenoble, France

**Keywords:** kidney biopsy, Zinder, glomerular diseases, tubulointerstitial changes, macroscopic hematuria, Niger

## Abstract

Kidney biopsy (KB) has become essential in the nephrologist’s approach to kidney diseases, both for diagnosis, treatment, and prognosis. Our objective is to describe the preliminary results of KBs in Niger, one of the poorest countries in the world. This is a descriptive cross-sectional study that took place over 36 months in the nephrology/dialysis department of the Zinder National Hospital. Biopsy results were obtained in less than 5 working days. Patients were responsible for covering the cost of the kidney biopsy. The data collected were analyzed using Epi Info V7 software. We performed 120 kidney biopsies during the study period. The average age of the patients was 35 years ± 15.4 [5–68]. The male/female sex ratio was 2:1. Patients’ medical history included herbal medicine use in 33% of cases and high blood pressure in 27.5% of cases. Proteinuria was present at a rate of ≥3 g/24 h in 46.6% of them. The primary indication for kidney biopsy was glomerular syndrome in 62.5% of cases, including 50% with nephrotic syndrome. All kidney biopsies were performed with real-time ultrasound guidance, using an automatic gun fitted with a 16G needle. Regarding complications, macroscopic hematuria was present in 12.5% of cases. Inadequate kidney biopsy was infrequent (5.8% of cases). The most common findings were (i) glomerular diseases (58.4%), such as membranoproliferative glomerulonephritis (13.3%), focal-segmental glomerulosclerosis (10.6%), lupus nephritis (8.8%), minimal change disease (8%), and membranous nephropathy (2.7%), and (ii) tubulointerstitial changes (31.8%). Diabetic nephropathy was rare (2.6%), as was IgA nephropathy (0.9%). We have demonstrated that implementing a sustainable kidney biopsy program in a very poor country is feasible, thanks to the dedication of a specialized renal pathologist. Having a clear diagnosis can assist in properly treating these renal patients according to international guidelines, thereby delaying the progression to end-stage kidney disease.

## 1. Introduction

Kidney needle biopsy is the removal of one (or more) small pieces of kidney tissue, called a kidney core biopsy, for histological and immunohistochemical analyses, used for diagnostic and follow-up purposes [1].

First described by Iversen and Brun in 1951, kidney puncture biopsy has significantly improved over the past two decades, becoming simpler and safer with the use of semi-automatic and automatic “guns”, as well as ultrasound assistance guidance. The contribution of renal puncture biopsy in diagnosing, evaluating, and selecting the therapy of nephropathy is substantial: it provides a precise diagnosis, assesses the extent of damage, suggests a prognosis, and aids in adapting therapies [1,2,3].

The kidney biopsy (KB) is performed in three stages: a preparation phase, an operational phase, and a monitoring phase. However, despite improvements in the technique, severe complications arising from kidney biopsy remain, primarily related to bleeding. Most of these complications occur within the first 24 h, with a favorable outcome for the majority of cases [4,5].

Niger, a developing country, is one of the poorest in the world (https://hdr.undp.org/data-center/human-development-index#/indicies/HDI, 20 January 2024). To date, Niger lacks a national kidney disease registry. Additionally, for a population of 24,000,000 inhabitants, there are very few nephrologists (*n* = 10) and only three chronic hemodialysis centers treating around 300 patients with end-stage kidney diseases. Until a few years ago, the kidney biopsy procedure was not available in Niger. This is the reason why in our University hospital, we decided in 2019 to implement the kidney biopsy procedure to identify kidney diseases that might be treatable.

The objective of this single-center retrospective study was to determine the epidemiological profile of patients who underwent kidney biopsies, identify the indications for kidney biopsies, evaluate their complications, and describe the diseases diagnosed as a result.

## 2. Materials and Methods

We conducted a descriptive cross-sectional study with retrospective data collection within the Nephrology-Dialysis department of the Zinder National Hospital (HNZ) in Niger, from November 2019 to November 2022. Since November 2019, our department has maintained a kidney biopsy registry, where all patients who underwent this procedure are recorded.

➢ Study population: This study included all patients admitted to the Nephrology-Hemodialysis Department for whom an indication of kidney biopsy was made during the study period.
➢ Inclusion criteria: All patients who underwent a biopsy of the native kidney at HNZ during the specified period were included.
➢ Exclusion criteria: Patients who had a kidney biopsy performed at the time of nephrectomy for tumor-related reasons were not included. We recorded the following information: sociodemographic, clinical, and paraclinical data of the patients, complications observed after kidney biopsy, and histological results.
➢ Kidney biopsies: Kidney ultrasound assessment was carried out just prior to the kidney biopsy in more than 50% of patients due to financial constraints. All kidney biopsies were percutaneous and performed using a BARD-type automatic gun with a single-use 16-gauge needle. Two tissue fragments were obtained—one for optical microscopy and one for immunofluorescence. The kidney biopsy cores were transported by land to Kano, Nigeria, through Maimoujia, a border town, to the Ultramedikx Pathology Laboratory. There, they were processed and then analyzed by a nephropathologist, Professor Atanda. After a delay of 72 to 96 hours, he sent us the report via email, which also included two to three photographs of the most demonstrative slides. All associated costs, including the biopsy procedure, transportation, histological examination, immunofluorescence staining, and the report from the nephropathologist, were borne by the patient, totalling CFA 135,000 or approximately EUR 206.
➢ Patient classification: The main syndromes were defined as follows:

*Nephrotic Syndrome*: In adults, nephrotic syndrome is defined by proteinuria equal to or greater than 3.5 g/24 h, hypoalbuminemia less than 30 g/L, and hypoprotidemia less than 60 g/L. In children, it is defined by proteinuria equal to or greater than 50 mg/kg/24 h, hypoalbuminemia below 25–30 g/L, and hypoprotidemia below 50–55 g/L.

*Rapidly Progressive Glomerulonephritis Syndrome*: This syndrome involves a rapid worsening of renal function including acute kidney injury (AKI) accompanied by initial macroscopic hematuria and proteinuria.

*Tubulointerstitial Nephropathy Syndrome*: This syndrome is characterized by regular proteinuria of less than 1 g/day without hypertension or edema, often associated with haematuria and AKI.

*Vascular Nephropathy Syndrome*: In this syndrome, high blood pressure is prominent, there are non-major abnormalities in the examination of the urinary sediment, but there is often severe and rapidly progressive renal insufficiency.

*Isolated Acute Kidney Injury (AKI):* AKI is defined as a rapid decline in the glomerular filtration rate (GFR), resulting in an increase in serum creatinine.

*Inadequate Kidney Biopsy:* A renal puncture biopsy is considered to be inadequate when the obtained fragment is not kidney tissue or contains fewer than 7 glomeruli.

*Minimal Proteinuria*: Proteinuria is considered to be minimal if it is less than 1 g/24 h.

*Average Proteinuria:* Proteinuria is categorized as average if it falls between 1 and 3 g/24 h.

*Abundant Proteinuria:* Proteinuria is considered to be abundant if it is equal to or greater than 3 g/24 h.

*Chronic Kidney Failure*: Chronic kidney failure is characterized by a reduction in functional renal mass, leading to a permanent reduction (for at least 3 months) in GFR below 60 mL/min/1.73 m^2^. This condition involves an irreversible reduction in the number of functional nephrons.

*Hematuria*: Macroscopic or microscopic hematuria were assessed within 24 h following the percutaneous kidney biopsy.

Statistical analyses

The data collected were analyzed using Epi Info V7 software. Results are presented as mean ± SD.

## 3. Results

During the study period, 400 indications for kidney biopsies were made: the patients had an average age of 33 ± 15.7 years; there was a male predominance with a sex ratio [M/F] of 1.94. However, due to cost constraints only 120 kidney biopsies were performed. For those who had a kidney biopsy, the age was of 35 ± 15.4 years (ns), with age ranging from 5 to 68 years, and there was a male predominance with a sex ratio [M/F] of 2 (ns). Regarding medical history, 30.8% (*n* = 37) of patients regularly used herbal medicine, 33.5% (*n* = 39) had chronic hypertension and were under medical treatment, and 6.7% (*n* = 8) were diabetic. Table 1 describes the patients’ distribution according to estimated glomerular filtration rate, whereas Table 2 describes the patients’ distribution according to 24-h proteinuria flow rate. The primary indication for kidney biopsies was glomerular syndrome in 62.5% of cases, including 50% with nephrotic syndrome (see Table 3). Among the 120 patients, 46.7% had nephrotic range proteinuria, 40.8% had proteinuria between 1 and 3 g/24 h, and 12.5% had proteinuria of less than 1 g/24 h. In addition, 12 were presenting with acute kidney injury (10%) (see Table 3).

A total of 54 patients (45%) underwent renal ultrasound before the kidney biopsy, which showed no contraindications to the procedure. For the other 66 patients, ultrasound was performed in the imaging room of the National Hospital of Zinder where the kidney biopsies were carried out. A total of 21 patients experienced complications following the kidney biopsies, including 15 cases of macroscopic hematuria and 6 cases of microscopic hematuria (see Table 4). All kidney biopsies were examined using light microscopy and immunofluorescence. Histologically, glomerular nephropathy was the dominant finding in 58.4% of cases (*n* = 66), followed by tubulointerstitial nephropathy in 31.5% of cases (*n* = 36), and vascular nephropathy in 7.3%. (*n* = 8). No specific lesion was found in 2.8% of cases (*n* = 3) (see Table 5).

It is clear than more than half of the patients had impaired kidney function (eGFR < 60 mL/min), accounting for 64% of cases (n = 77).

The initial histopathology diagnosis revealed chronic tubulointerstitial nephritis as the most prevalent (19.5%), followed by membranoproliferative glomerulonephritis (MPGN) (13.3%), and focal and segmental glomerulosclerosis (FSGS) (10.6%). Non-specific chronic glomerulonephritis was detected in 11% of the cohort. Pre-kidney biopsies (KBs) echography also identified two renal tumors, accounting for 1.7% of the cases (one case of Burkitt lymphoma and one case of renal cell carcinoma).

Of the 12 patients presenting with acute kidney injury, 7 presented with acute tubular necrosis, 3 with acute interstitial nephritis, and 2 with post-infectious glomerunephritis. Regarding the latter 2 patients, these required dialysis support but refused and subsequently one died and the other was lost to follow-up. All the others recovered except for one patient with acute interstitial nephritis for whom the last serum creatinine was at 300 μmol/L. 

**Figure 1:**
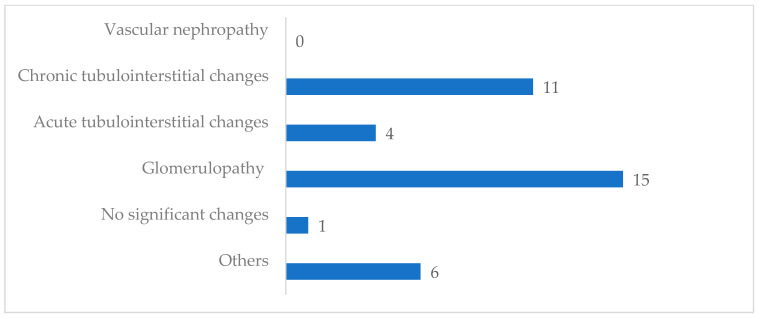
Pathological findings in renal biopsies from patients that have been taking herbal medicine.

With regard to the patients that were taking phytotherapy prior to the kidney biopsy (n = 37), renal pathology showed glomerular lesions in 15 (40.5%) and tubulointerstitial changes in 15 (40.5%) (see Figure 1). Indeed, amongst 22 patients presenting with chronic tubulointerstitial changes 11 were taking phytotherapy; for those presenting with acute tubulointerstitial changes (n = 7), 4 (57.1%) were undergoing phytotherapy.

## 4. Discussion

The practice of renal puncture biopsy in clinical nephrology is underdeveloped in many developing countries, especially in Africa. The Nephrology-Dialysis Department of the Zinder National Hospital is the second center in the country, following Niamey (the capital of Niger), and the very first to undertake this practice in Niger. We performed 120 percutaneous kidney biopsies over a 3-year period, from November 2019 to November 2022.

This study faced several challenges, including the lack of renal pathologists in Niger, the relatively high cost borne by the patients, and the need to find a location to send kidney biopsies for processing and receive results within a reasonable timeframe. Although as many as 400 patients could have benefitted from kidney biopsy procedures during the study period, only 120 (30%) did so due to financial constraints.

Indeed, in Western Africa and in sub-Saharan countries there are very few renal pathologists, and very few nephrology departments are able to perform percutaneous kidney biopsies, whereas it is very common practice in countries in the Maghreb. For example, in Senegal a study reports on a series of 42 lupus nephritis patients of whom only 53.38% have had a kidney biopsy [6]. Nigeria is the most populous country in Africa, with more than 213 million inhabitants. Percutaneous kidney biopsies are performed in Nigeria, but there are very few studies on that subject [7,8]. They deal either with nephrotic syndrome in a pediatric population or with HIV-infected patients.

In our study, an average of 40 kidney biopsies were performed per year, a significantly high rate compared to reports from nephrology centers in Morocco (23 KBs per year) and in the French Antilles (27 per year) [9,10]. In contrast, Ben Salem et al. in Tunisia reported in their center a higher frequency of 71.6 KBs/year [11].

The average age of patients was 35 years, with extremes of 5 and 68 years. The most represented age group was [15–34], making up 48.33% of the cohort. This average age was lower than that reported in studies conducted by Ben Salem et al. in Tunisia in 2020 (45 years old) [11] or in a study carried out in a military hospital in Morocco (44 years old) [12], with a standard deviation of 16 and 15 years, respectively. A study carried out by N’Dah et al. found an average age of 32.9 ± 13.8 years [13]. These differences could be attributed to the relatively youthful population in Niger and Mali compared to those in Tunisia or Morocco [14,15,16,17]. Our sample consisted of 80 men (66.7%) and 40 women (33.3%), resulting in a sex ratio of 2:1. This male predominance aligns with data from other studies [12,18,19] although some studies report a female predominance [10,20].

In our patient cohort, we observed that 33.3% had used herbal medicine, while 32.5% had hypertension, and 6.7% and 1.7% had type 2 diabetes and sickle cell disease, respectively. In a study by Mhamedi et al., from Morocco, 17% of patients had hypertension, and lupus was present in 14% of patients, while glomerulopathy was found in 11% of cases [21].

Traditional treatment or herbal medicine is a common practice worldwide, especially in Africa. A cross-sectional study on the use of herbal medicine was conducted in the Nephrology department of the University Hospital of Fes (Morocco): it was found that amongst 471 patients, the prevalence of herbal medicine use was as high as 50.7%. The most commonly used herbal medicine were Rosmarinus officinalis L.; Origanum compactum Benth; Artemisia herba-alba Asso and Mentha pulegium L; however, potentially harmful herbal medicine included Aristolochia longa [22]. In a study from northern Cameroon (close to the Niger border), longstanding use of herbal medicine as well as street medications was acknowledged by 90.9%, and 87.5% of CKD patients, respectively [23]. Likewise, in a large study from Ghana with almost 2800 participants independent predictors of CKD included herbal medications (aOR 1.39 (1.10–1.75)) [24]. The use of herbal medicine might explain the higher incidence of tubulointerstitial damage in our patients. Patients often resort to traditional remedies before seeking medical advice, only turning to healthcare professionals when their condition fails to improve or worsens [25,26].

The primary indication for kidney biopsy in our patients was nephrotic syndrome (50.0% of cases). In Mhamedi et al.’s study, nephrotic syndrome also dominated as the primary indication of KB (60%) across all age groups; the presence of a systemic disease with proteinuria or renal involvement (14%) constituted the second most common indication for kidney biopsy, alongside acute kidney injury at 14%. Following close were glomerular syndrome (6%) and rapidly progressive glomerulonephritis (3%) [21]. Within our series, we encountered 21 cases of complications, which included 12.5% (n = 15) of cases of macroscopic hematuria and 5% (n = 6) of cases of microscopic hematuria (detected by an urine dipstick) among the set of KBs. The absence of perirenal hematoma or arteriovenous fistula could be attributed to the fact that we do not routinely carry out renal ultrasound following the biopsy due to cost issues. The relatively high rate of macroscopic hematuria in our study may be attributed to the relatively small size of our study sample.

Lefaucheur et al. reported that several factors were associated with the risk of hemorrhagic complications following kidney biopsy [27]. This included the sex, i.e., women being at a higher risk, and the age, i.e., the risk was elevated for individuals at both age extremes (<20 years and >70 years), regardless of the histological diagnosis. 

In 2004, Alebiosu and Kadiri reported on a single-center experience in Nigeria on percutaneous kidney biopsy as an outpatient procedure [28]. More recently, Obiagwu et al. have reported that a percutaneous kidney biopsy can be safely carried out as an out-patient procedure in children. However, low hemoglobin concentration was the major risk factor for complications [29]. A recent systematic review addressed complications associated with kidney biopsy in low- and middle-income countries: they were found to be low, and were comparable to those in other settings, and occurred more sparingly when real-time ultrasound techniques or automated kidney biopsy needles are used [30]. This suggests the need to expand the use of this procedure to improve diagnosis of kidney pathologies and the choice of therapy when indicated. 

In our study, glomerular nephropathies were prevalent at 58.4%, with primary nephropathy surpassing secondary cases, accounting for 46.9% and 11.5%, respectively. This observation was consistent with findings by N’Dah et al. in Mali [13] and En-niya et al. in Morocco [31], where they also observed a predominance of primary glomerular nephropathy over secondary cases. Among primary glomerular nephropathy, membranoproliferative glomerulonephritis was the most common, occurring in 13.3% of cases. In contrast, Traore et al.’s study [10] found focal segmental hyalinosis to be dominant in 16% of cases and a predominance of minimal glomerular lesions in Mbarki et al.’s study [20] in 13.7% of cases. Tubulointerstitial nephropathy in our study represented 31% of all histological lesions diagnosed, with a predominance of chronic tubulointerstitial nephropathy and acute tubular necrosis at 20% and 6.2%, respectively. These findings differ from those of Ben Salem et al. in Tunisia, Mbarki et al. in Morocco and N’Dah et al. in Mali, where they reported rates of tubulointerstitial nephropathy of 10.3% [11], 3.2% [20] and 2.2% [13], respectively.

This variance could be attributed to the behavior of our population when confronted with the phenomenon of “anarchic” herbal medicine and self-medication, as these factors are the primary contributors to tubulointerstitial nephropathy.

We found only three cases of diabetic nephropathy. Indeed, no one knows the prevalence of type 2 diabetes in Niger. However, in our nephrology clinic diabetic patients represent barely 5% of the overall CKD patients. A recent cross-sectional study has assessed diabetes 2D prevalence and risk factors in a rural Malian community (n = 412 subjects). It was found that its overall prevalence was 7.5%, and risk factors were age, family history of diabetes, hypertension, waist circumference, and fetal macrosomia. Notably, 61.3% of type 2 diabetic subjects were unaware of their diabetic status before the study [32]. Recently, a systematic review and meta-analysis has found that the overall prevalence of both overweight and obesity was 61.4% amongst type 2 diabetic African patients. However, there were big disparities across Africa, i.e., the pooled prevalence of both overweight and obesity across the five geographical areas in Africa ranged from 56.9% in East Africa to 88.5% in Southern Africa [33]. In addition, a recent systematic review has demonstrated that the rates of retention in care of people living with diabetes are poor in sub-Saharan countries [34].

## 5. Conclusions

The present study on percutaneous renal biopsy at the Zinder National Hospital (Niger) aimed to include 400 patients, but ultimately only 120 were able to undergo the procedure. The objective of this study is to describe the results of the initial biopsies performed in Niger. These first biopsies have contributed to clarifying the diagnosis of glomerular syndromes in relatively young patients with a history of herbal medicine, hypertension, and diabetes. This study demonstrates that renal biopsy is a well-controlled procedure, with no major complications observed, except for a few cases of manageable hematuria. This work confirms the significance of kidney biopsy in clinical nephrology as it has helped confirm diagnoses of glomerular lesions, with membranoproliferative glomerulonephritis predominating, followed by focal and segmental glomerulosclerosis (FSGS), along with tubulointerstitial nephropathy.

We conclude that the practice of kidney biopsy has been successfully implemented at the Zinder National Hospital (Niger). While awaiting its expansion to other nephrological centers in Niger, further efforts are needed, particularly in the on-site interpretation of kidney biopsy results and in making the procedure financially accessible for most of the patients for whom it is necessary.

## Figures and Tables

**Table 1:** Distribution of patients according to glomerular filtration rate.

Glomerular Filtration Rate (GFR); mL/min	Number of Patients	Percentage%
≥90	29	24.2
[60–89]	14	11.6
[30–59]	13	10.8
[15–29]	11	9.2
<15	53	44.2
Total	120	100.0

**Table 2:** Distribution of patients according to 24-h proteinuria flow rate.

24-h Proteinuria	Number of Patients	Percentage%
Abundant	56	46.7
Minimal	15	12.5
Average	49	40.8
Total	120	100.0

**Table 3:** Distribution of patients according to kidney biopsy (KB) indication.

Indication of KB	Number of Cases	Percentage%
Unexplained Hematuria	1	0.83
Unexplained Chronic Kidney Failure	35	29.17
Lupus	6	5.0
Massive Non-Nephrotic Proteinuria	1	0.83
Scabiosis	2	1.67
Glomerular Syndrome	15	12.5
Nephrotic Syndrome	60	50.0
Total	120	100.0
Acute kidney injury (with some of the above symptoms)	12	10

**Table 4:** Post-kidney biopsy complications.

Type of Complications	Number of Cases	Percentage%
Gross hematuria	15	71.4
Microscopic Hematuria	6	28.6
Total	21	100.0

**Table 5:** Distribution of patients according to histological diagnoses.

Types of Lesions	Number of Patients	Percentage%
Minimal Change Disease	9	8.0
Focal And Segmental Glomerulosclerosis	12	10.6
Membranous Glomerulonephritis	3	2.7
Diffuse Glomerulosclerosis	10	8.8
Membranoproliferative Glomerulonephritis	15	13.3
Iga Nephropathy	1	0.9
Lupus Nephropathy	10	8.8
Diabetic Nephropathy	3	2.7
Vascular Nephropathy	8	7.1
Chronic Tubulointerstitial Nephritis	22	19.5
Acute Tubular Necrosis	7	6.2
Acute Tubulointerstitial Nephritis	1	0.9
Acute Interstitial Nephritis	2	1.8
Burkitt’sLymphoma	1	0.9
Chronic Pyelonephritis	2	1.8
Renal Cell Carcinoma	1	0.9
Post-Infectious Glomerunephritis	2	1.8
Nodular Glomerulosclerosis	1	0.9
No Damage	3	2.7
Total	113	100.0

PS: Of the 120 kidney biopsies performed, 7 were inadequate (non-renal tissue).

## Data Availability

The data presented in this study are available on request from the corresponding author.
